# Crystal structure analysis, overexpression and refolding behaviour of a DING protein with single mutation

**DOI:** 10.1107/S0909049513020694

**Published:** 2013-09-29

**Authors:** Zuoqi Gai, Akiyoshi Nakamura, Yoshikazu Tanaka, Nagisa Hirano, Isao Tanaka, Min Yao

**Affiliations:** a Faculty of Advanced Life Sciences, Hokkaido University, Sapporo 060-0810, Japan; b Graduate School of Science, Hokkaido University, Sapporo 060-0810, Japan

**Keywords:** DING, HPBP, crystal structure, overexpression, refolding

## Abstract

After crystallization of a certain protein–RNA complex, well diffracting crystals were obtained. However, the asymmetric unit of the crystal was too small to locate any components. Mass spectrometry and X-ray crystal structure analysis showed that it was a member of the DING protein family (HPBP). Surprisingly, the structure of HPBP reported previously was also determined accidentally as a contaminant, suggesting that HPBP has a strong tendency to crystallize. Furthermore, DING proteins were reported to relate in disease. These observations suggest that DING has potential for application in a wide range of research fields. To enable further analyses, a system for preparation of HPBP was constructed. As HPBP was expressed in insoluble form in *Escherichia coli*, it was unfolded chemically and refolded. Finally, a very high yield preparation method was constructed, in which 43 mg of HPBP was obtained from 1 L of culture. Furthermore, to evaluate the validity of refolding, its crystal structure was determined at 1.03 Å resolution. The determined structure was identical to the native structure, in which two disulfide bonds were recovered correctly and a phosphate ion was captured. Based on these results, it was concluded that the refolded HPBP recovers its structure correctly.

## Abstract 2

Crystals of a member of the DING protein family (HPBP) were obtained accidentally, and the structure was determined at 1.35 Å resolution. For further analysis, a system for preparation of HPBP was constructed and the structure of a prepared sample was confirmed by X-ray crystal structure analysis at 1.03 Å resolution.

## 1. Introduction

DING is a family of proteins with molecular mass of approximately 40 kDa, with a conserved N-terminal sequence of DINGGG. DING proteins have been identified in a wide range of organisms: prokaryotes, animals, fungi and plants (Berna *et al.*, 2008 ▶). They have attracted attention due to recent reports that they are involved in many diseases, including rheumatoid arthritis, lithiasis, atherosclerosis, some tumours, tumour-associated cachexia and bacterial and viral infections (Berna *et al.*, 2009 ▶; Bookland *et al.*, 2011 ▶; Lesner *et al.*, 2009 ▶). However, their relevance and/or biological functions have yet to be elucidated. Neither a gene nor ORF encoding DING proteins have yet been identified in eukaryotes despite the high frequency of discovery in eukaryotes (Berna *et al.*, 2008 ▶), which has hampered investigation of eukaryotic DING proteins. In contrast, genes of DING proteins have been identified in bacteria (Scott & Wu, 2005 ▶).

DING proteins are one of three families within a superfamily of bacterial phosphate-binding proteins (Zhang *et al.*, 2007 ▶). To date, the crystal structures of two DING proteins, *i.e.* human phosphate-binding protein (HPBP) and DING protein from *Pseudomonas fluorescens* SBW25 (*Pfl*DING), have been reported (Morales *et al.*, 2006 ▶; Liebschner *et al.*, 2009 ▶). In both structures, a phosphate ion was captured in a binding cleft between two domains. For *Pfl*DING, a heterologous overexpression system was constructed using *Escherichia coli* (Scott & Wu, 2005 ▶), and the phosphate-binding mode was precisely elucidated based on the sub-angström resolution structure of the recombinant *Pfl*DING protein (Liebschner *et al.*, 2009 ▶). Furthermore, functional analysis was also carried out based on the structural information (Ball *et al.*, 2012 ▶). It should be noted that the first crystal of HPBP was accidentally grown from a paraoxonase 1 solution purified from plasma (Morales *et al.*, 2006 ▶). From this serendipitous discovery, the authors determined the amino acid sequence by combining the X-ray structure and mass spectrometry (Diemer *et al.*, 2008 ▶). Moreover, HPBP was reported to inhibit HIV-1 gene transcription and replication (Cherrier *et al.*, 2011 ▶). However, as a system for overexpression has not been reported for HPBP, biochemical experiments such as mutation analysis or domain truncation have not been possible. To gain further understanding of this protein, it is necessary to construct a heterologous overexpression system.

In the present study, HPBP with a single mutation was unexpectedly crystallized during a crystallization trial of a certain protein–RNA complex, as was the case for the first crystal of HPBP. For further analysis of HPBP, an overexpression and refolding system was constructed. The obtained protein was evaluated by X-ray crystallography, and the results indicated that HPBP was correctly refolded to the native structure. To our knowledge, this is the first report of a system for overproduction of HPBP as well as refolding of DING proteins.

## 2. Experimental procedures

### 2.1. Crystallization of HPBP

HPBP with single mutation was unexpectedly crystallized during a crystallization trial of a protein–RNA complex. A protein–RNA complex composed of proteins of 53 kDa, 52 kDa and 11 kDa, and its substrate aminoacyl-tRNA were prepared as described previously (Nakamura *et al.*, 2006 ▶; Curnow *et al.*, 1997 ▶). The purified protein complex and the substrate aminoacyl-tRNA were crystallized and small crystals were grown by the sitting-drop vapour-diffusion method in 100 m*M* Tris-HCl buffer, pH 8.0, 16% polyethylene glycol 8000, 100 m*M* NaCl.

### 2.2. Mass spectrometry

Crystals were dissolved in water and then subjected to SDS-PAGE. The band was cut out from the gel and treated with trypsin. The extracted fragments were analysed by LC-MS (LXQ linear ion trap; Thermo Scientific, Waltham, MA, USA) coupled with a Magic2002 (Michrom BioResources, Auburn, CA, USA) and nanospray electrospray ioniser (ESI; AMR, Tokyo, Japan). The determined molecular masses were analysed by *Mascot* search (Matrix Science, London, UK).

### 2.3. Preparation and crystallization of recombinant HPBP

The amino acid sequence of recombinant HPBP (rHPBP) was determined from the electron density map of the HPBP crystal described above, in which the reported sequence was used as a reference (Morales *et al.*, 2006 ▶). The gene of rHPBP synthesized by Genscript (Piscataway, NJ, USA) was cloned into the NcoI and BamHI sites of the pET28b vector (EMD Biosciences, Temecula, CA, USA). A transformed *E. coli* strain, B834 (DE3), harbouring HPBP expression vector, was grown at 310 K in LB medium until the early stationary phase, and then isopropyl β-d-thiogalactopyranoside was added to a final concentration of 0.5 m*M* to induce expression of the target protein. The culture was grown for an additional 18 h at 298 K. Cells were disrupted using a sonicator (Branson, Danbury, CT, USA) in PBS (100 m*M* Na_2_HPO_4_, 18 m*M* KH_2_PO_4_, 137 m*M* NaCl and 27 m*M* KCl). The cell lysate was centrifuged at 12000 × *g*, 277 K, for 30 min and the insoluble fraction was collected. The inclusion body of rHPBP was purified by re-suspending with PBS containing 0.25% Triton X-100 and centrifugation three times. The obtained inclusion body was rinsed three times with PBS, and stored at 243 K.

The purified inclusion body was solubilized by incubation with denaturing buffer (6 *M* guanidinium hydrochloride, 20 m*M* sodium phosphate, pH 8.0, 200 m*M* NaCl, 2 m*M* EDTA and 1 m*M* β-mercaptoethanol) for 18 h at room temperature, followed by centrifugation. The unfolded rHPBP was refolded by diluting 1000-fold with refolding buffer (20 m*M* sodium phosphate, pH 8.0, 200 m*M* NaCl, 400 m*M* l-arginine hydrochloride and 1 m*M* EDTA) followed by incubation with stirring for 18 h at 277 K. After concentrating, the refolded rHPBP solution was dialysed against 20 m*M* sodium phosphate (pH 8.0), 200 m*M* NaCl and then 20 m*M* Tris-HCl (pH 9.0). The soluble fraction was loaded onto a 10 ml HiTrap QXL column (GE Healthcare Bio-Sciences AB, Uppsala, Sweden). The flow-through fraction was collected, and then further purified on a HiLoad 26/60 Superdex 200-pg column (GE Healthcare Bio-Sciences). The purified rHPBP was dialysed against 20 m*M* HEPES (pH 7.6), 10 m*M* MgCl_2_, 5% glycerol and 1 m*M* β-mercaptoethanol, and then concentrated to 8.2 mg ml^−1^. Crystals of rHPBP were grown in the same conditions under which crystals of HPBP were crystallized as a contaminant.

### 2.4. X-ray diffraction experiments and structure determination

X-ray diffraction patterns of HPBP and rHPBP were obtained on the beamlines BL17A and BL5A, respectively, at the Photon Factory (Tsukuba, Japan). The diffraction data were indexed, integrated, scaled and merged using the *XDS* package (Kabsch, 2010 ▶). Crystallographic parameters are summarized in Table 1 ▶. The crystal structure of native HPBP was determined by the molecular replacement method using the reported HPBP structure [Protein Data Bank (PDB) ID 2v3q] as a search model with the program *MOLREP* (Vagin & Teplyakov, 1997 ▶). The structure of rHPBP was determined from the structure of native HPBP. Throughout the refinement in both native HPBP and rHPBP, reflections of the index same as those used for calculation of the *R*_free_ factor for the reported HPBP structure were used for *R*_free_ calculation. The positional and individual *B*-factor refinements were carried out with the program *PHENIX* (Adams *et al.*, 2010 ▶). The structure was modified manually with the program *COOT* (Emsley & Cowtan, 2004 ▶). The *R*_work_/*R*_free_ factors converged to 0.1484/0.1696 for native HPBP and 0.1735/0.1840 for rHPBP. The refinement statistics are summarized in Table 1 ▶. The coordinates have been deposited in the PDB (IDs 3w9w and 3w9v).

## 3. Results

### 3.1. Crystallization and identification of HPBP

**Figure 1:**
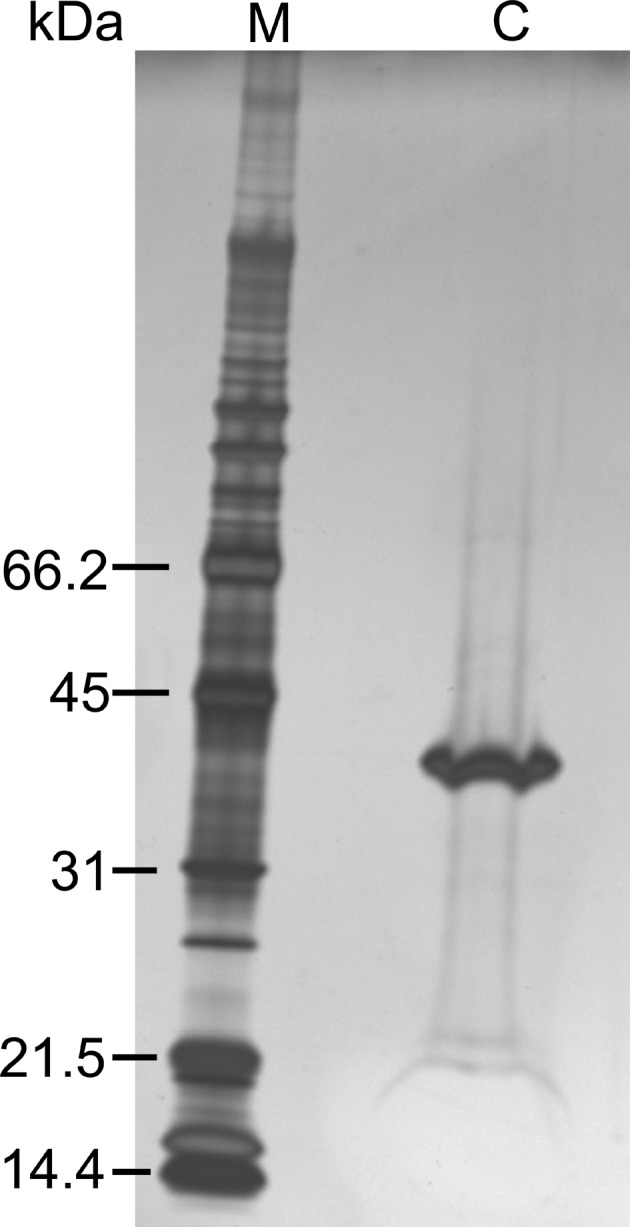
Silver stain SDS-PAGE of the obtained crystals. M and C represent marker and the crystal of native HPBP, respectively.

**Figure 2:**
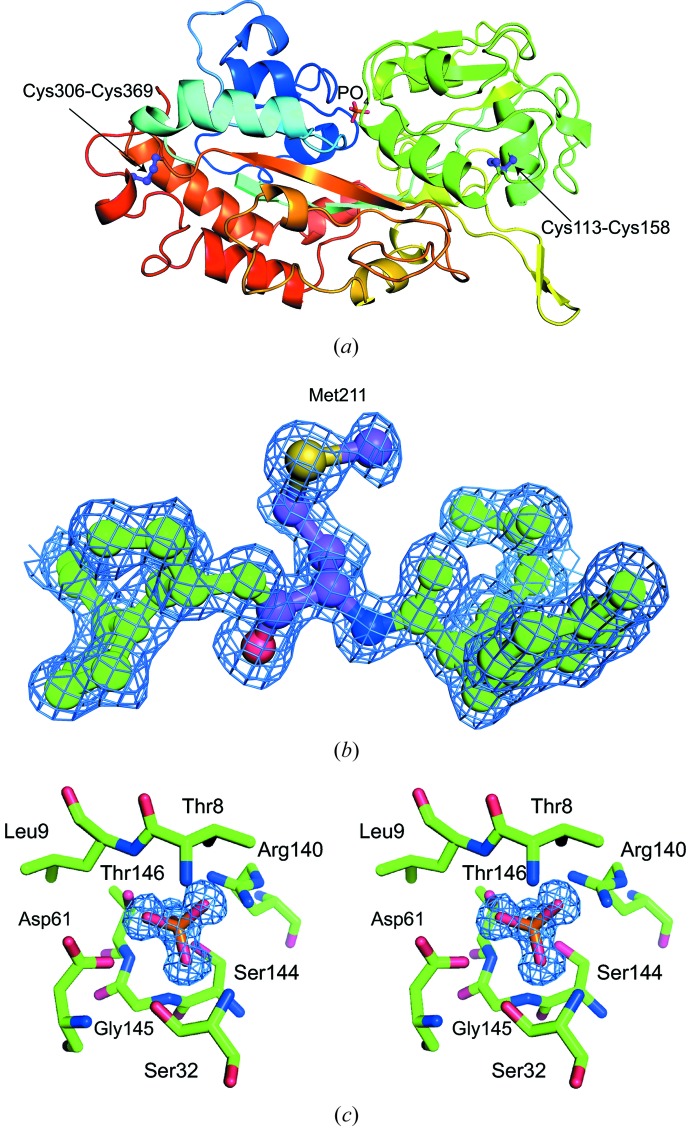
Crystal structure of native HPBP. (*a*) Ribbon diagram. The model is coloured according to the sequence from blue at the N-terminus to red at the C-terminus. Two disulfide bonds and phosphate are also shown. (*b*) Electron density of the substituted Met211 residue. (*c*) Phosphate binding site (stereoview).

We attempted to crystallize a protein complex composed of proteins of 53 kDa, 52 kDa and 11 kDa in complex with its substrate. After crystallization trials and optimization, small crystals were obtained, and a high-resolution X-ray diffraction dataset (1.35 Å resolution) was collected. The crystal belonged to space group *C*2 with unit-cell parameters *a* = 94.3 Å, *b* = 87.1 Å, *c* = 88.6 Å, β = 90.82°. However, the calculated asymmetric unit (727644 Å^3^) was too small to locate any of the components. The results of SDS-PAGE of these crystals indicated a protein with a molecular mass of approximately 40 kDa that corresponded to none of the target proteins (Fig. 1 ▶). To identify the observed protein, it was digested with trypsin and the resultant fragments were analysed by LC-MS. *Mascot* search of the obtained molecular masses suggested HPBP, a member of the DING protein family, as a candidate. Next, a PDB search was performed for crystal structures with unit-cell parameters similar to those of the obtained crystal. Surprisingly, HPBP (space group *C*222_1_, *a* = 96.3 Å, *b* = 86.8 Å, *c* = 89.9 Å) was identified again. It should be noted that the reported HPBP was crystallized as a protein contaminant of paraoxonase 1 purified from human serum (Morales *et al.*, 2006 ▶). These observations strongly suggest that the crystal would be HPBP contaminant. Therefore, we attempted to solve the structure by molecular replacement using the reported HPBP (PDB ID 2v3q) as a search model. As expected, the structure was determined (hereafter designated as the native HPBP structure) (Fig. 2*a* ▶). The clear electron density revealed that the amino acid sequence was identical to that of reported HPBP except for one residue, *i.e.* Ile211 was substituted by Met (Fig. 2*b* ▶). As observed for the HPBP reported previously (Morales *et al.*, 2006 ▶), a phosphate ion was captured in the binding pocket using T8, L9, S32, D61, R140, S144, G145 and T146 (Fig. 2*c* ▶). In addition, two disulfide bonds were formed between Cys113 and Cys158 and between Cys306 and Cys369 (Fig. 2*a* ▶).

### 3.2. Refolded recombinant HPBP and its structure

**Figure 3:**
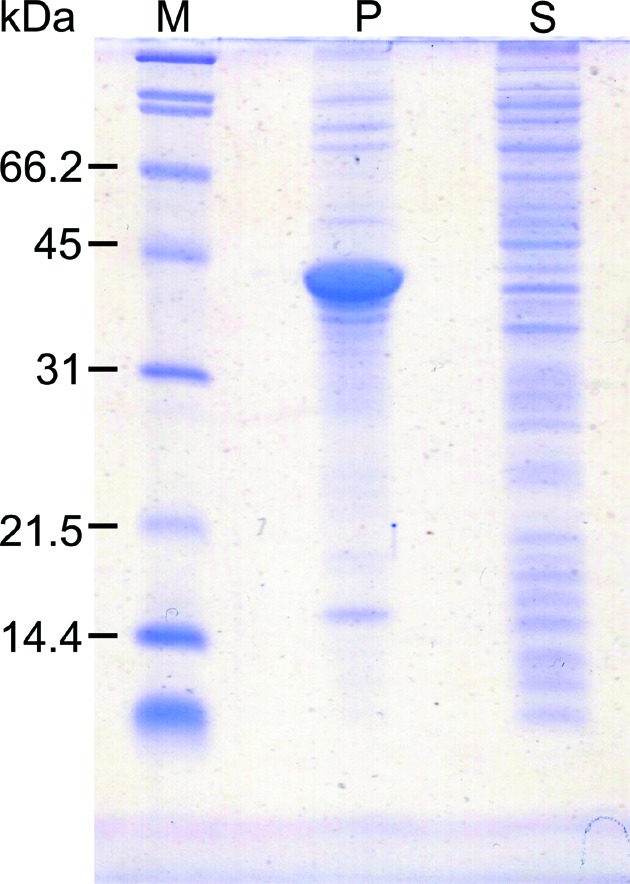
SDS-PAGE of rHPBP expressed by *Escherichia coli.* M is protein marker, P and S represent precipitant and supernatant of recombinant HPBP, respectively.

To enable further study of DING proteins, an *E. coli* expression system for HPBP was constructed. The recombinant HPBP (rHPBP) was overexpressed as an insoluble protein (Fig. 3*a* ▶). Then, the insoluble rHPBP was unfolded with 6 *M* guanidinium hydrochloride and 1 m*M* β-mercapto­ethanol. The chemically unfolded rHPBP was refolded by the dilution method. The refolding efficiency was markedly improved by adding 400 m*M* l-arginine hydrochloride. The refolded rHPBP was further purified by chromatography, and the final yield was 43 mg per litre of culture.

**Figure 4:**
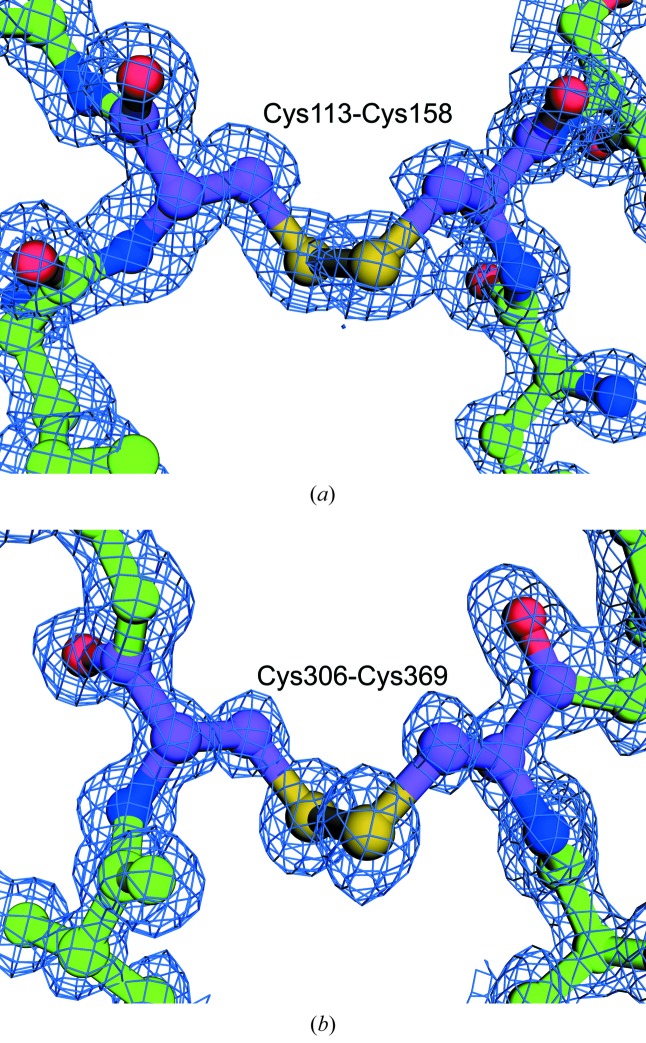
Crystal structure of refolded rHPBP. Disulfide bonds between Cys113 and Cys158 (*a*) and Cys306 and Cys369 (*b*).

To evaluate whether rHPBP was refolded correctly, the purified rHPBP was crystallized and its structure was determined. The structure of rHPBP was determined at a resolution of 1.03 Å. The revealed structure was well superposed on the native HPBP with r.m.s.d. of 0.04 Å for 327 Cα atoms. A phosphate ion was captured at the identical site to native HPBP, with identical residues participating in recognition. Furthermore, two disulfide bonds were formed correctly (Fig. 4 ▶). Based on these observations, it was concluded that rHPBP was correctly refolded to the native structure.

## 4. Discussion

The crystal structure of contaminating HPBP has been determined twice, *i.e.* by Morales *et al.* (2006 ▶) and by our group. In addition, the crystal structure of another DING protein was determined at sub-angström resolution (0.88 Å) (Liebschner *et al.*, 2009 ▶). These observations suggest that DING proteins may have a strong tendency to crystallize. Furthermore, DING proteins have attracted attention as they were reported to relate to numerous diseases (Berna *et al.*, 2009 ▶; Bookland *et al.*, 2011 ▶). These observations suggest that DING has the potential for application as crystallization-enhancing fusion protein and/or development of therapeutic agent for some disease as target protein or candidate. However, the nucleotide sequence of eukaryotic DING has not yet been identified and an overexpression system has not been constructed, which has hampered investigation of eukaryotic DING proteins. The present study enabled preparation of rHPBP in very high yield (43 mg per litre of culture). This method is expected to accelerate further study of DING.

Crystals of the refolded rHPBP diffracted to high resolution (1.03 Å). Although there are many model proteins for X-ray crystallography that can be easily crystallized (*e.g.* lysozyme and trypsin), there are few refolded proteins which crystallize in high resolution. Therefore, HPBP has potential for application as a model protein for protein science. For this purpose, the outstandingly high yield would be a great advantage. We hope that this report will be helpful for a wide range of research.

The remaining issue to be considered is the source of HPBP. In the present study, the first target proteins were expressed by *E. coli*. In addition, the substrate aminoacylated-RNA was prepared using enzymes expressed by *E. coli*. However, no gene corresponding to the HPBP was found in the *E. coli* genome database. Therefore, the possibility of contamination from the expression host was excluded. It should be noted here that the reaction buffer used in order to prepare the substrate aminoacylated-tRNA contained BSA at a concentration of 0.1 mg ml^−1^. In the previous case, HPBP was purified from human plasma (Morales *et al.*, 2006 ▶). Taken together, it is plausible that HPBP was a contaminant in the BSA used for substrate preparation. However, the reaction solution was treated with phenol after the reaction, and therefore the contaminating HPBP should have been denatured. One probable explanation is that HBPB contaminant in BSA was denatured by phenol after aminoacylation reaction, but the tertiary structure was recovered during the subsequent procedures. This speculation is consistent with the very high yield of refolded rHPBP obtained in the present study.

## Supplementary Material

PDB reference: 3w9w

PDB reference: 3w9v

## Figures and Tables

**Table 1:** Crystal parameters and data collection statistics Values in parentheses are for the last resolution shell.

Data collection		
Crystal	Native HPBP	Refolded rHPBP
Space group	*C*2	*C*2
Beamline	PF BL17A	PF BL5A
Wavelength (Å)	1.0000	1.0000
Unit-cell parameter (Å)	*a* = 94.27, *b* = 87.14, *c* = 88.60, β = 90.82	*a* = 94.34, *b* = 86.89, *c* = 88.64, β = 90.81
Resolution range (Å)	43.57–1.35 (1.43–1.35)	44.32–1.03 (1.06–1.03)
No. of observed reflections	563219	1379676
No. of unique reflections	150208	346764
Completeness (%)	95.9 (85.5)	99.1 (94.7)
*I*/σ(*I*)	16.25 (3.21)	14.3 (2.08)
*R*_meas_	0.068 (0.456)	0.05 (0.72)
No. of residues	376	376

Refinement statistics		
Resolution range (Å)	43.57–1.35	29.54–1.03
No. of reflections (*R*_free_)	7462	17185
No. of water molecules	1368	1281
*R*/*R*_free_	0.1484/0.1696	0.1735/0.1840
R.m.s.d. bond lengths (Å)	0.006	0.004
R.m.s.d. angles (°)	1.059	1.041
Average *B*-factor (Å^2^)	11.73	12.59
Protein molecules	2	2
